# Supplementary material for: Monopole-like orbital-momentum locking and the induced orbital transport in topological chiral semimetals
Source: Proc Natl Acad Sci U S A. 2023 Nov 20;120(48):e2305541120. doi: 10.1073/pnas.2305541120 (PMC10691347; doi:10.1073/pnas.2305541120)
Supplement: Supplementary file 1 — Appendix 01 (PDF) [file pnas.2305541120.sapp.pdf]

Supporting Information for
Monopole-like orbital-momentum locking and the induced orbital transport in topological chiral semimetals

Qun Yang[a,b*], Jiewen Xiao[b], Iñigo Robredo[a,c], Maia G. Vergniory[a,c], Binghai Yan[b*], and Claudia Felser[a*]

[a]Max Planck Institute for Chemical Physics of Solids, 01187 Dresden, Germany

[b]Department of Condensed Matter Physics, Weizmann Institute of Science, Rehovot 7610001, Israel

[c]Donostia International Physics Center, 20018 Donostia-San Sebastian, Spain

[*]**Email:** Qun.Yang@cpfs.mpg.de (Q.Y.), binghai.yan@weizmann.ac.il (B.Y.), Claudia.Felser@cpfs.mpg.de (C.F.)

This PDF file includes:

Supporting text
Figures S1 to S7
Tables S1 to S3
SI References

Supporting Information Text

Symmetry properties for OHC and ME susceptibility tensor

The symmetry equivalent quantity of the OHC tensor ($\sigma_{\alpha\beta}^{\gamma}$) can be simply expressed as $O_{\alpha\beta}^{\gamma} = v_{\alpha}L_{\gamma}v_{\beta}$, where α, β, and γ represent the direction for the induced orbital current, applied electric field, and orbital polarization, respectively. The symmetry allowed $O_{\alpha\beta}^{\gamma}$ remains invariant under all symmetry operations of the material. Therefore, if $O_{\alpha\beta}^{\gamma}$ remains positive under a chosen symmetry operation, the corresponding component of $O_{\alpha\beta}^{\gamma}$ is symmetry-allowed. Otherwise, it will be forbidden by symmetry. For materials in SG $P2_13$, there are a total of 12 symmetry operations which are generated by twofold screw rotations 2_{1x} = {C_{2x} | 0.5, 0.5, 0}, 2_{1y} = {C_{2y} | 0, 0.5, 0.5} and diagonal threefold rotation $C_{3,111}$ = {$C_{3,111}$ | 0.0, 0.0, 0}. 2_{1x} or 2_{1y} symmetry take $O_{\alpha\beta}^{\gamma} = -O_{\alpha\beta}^{\gamma}$ when any two or three of the indices are identical. Therefore, $\sigma_{\alpha\beta}^{\gamma}$ is non-zero only when $\alpha \neq \beta \neq \gamma$. Furthermore, the additional $C_{3,111}$ symmetry which transforms three indices (x, y, z) to (y, z, x) makes the non-zero components relate each other as: $\sigma_{xy}^{z} = \sigma_{yz}^{x} = \sigma_{zx}^{y}$ and $\sigma_{yx}^{z} = \sigma_{zy}^{x} = \sigma_{xz}^{y}$. By similar symmetry analysis, the symmetry properties of OHC tensor for all chiral structures in 11 chiral point groups can be obtained as shown in Table S2.

While for the ME susceptibility tensor (a_{ij}), its symmetry equivalent quantity can be expressed as $A_{ij} = m_i v_j$. Here, m_i and v_j are the induced magnetization along i-direction and band velocity in j-direction. 2_{1x} or 2_{1y} symmetry take A_{ij} to $-A_{ij}$ for $i \neq j$. Therefore, all the off-diagonal elements of a_{ij} have to vanish. The diagonal elements with $i = j$ are non-zero and have to be equal because of the additional $C_{3,111}$ symmetry. The symmetry properties of ME susceptibility tensor for all chiral structures in 11 chiral point groups can be referred from reference (1).

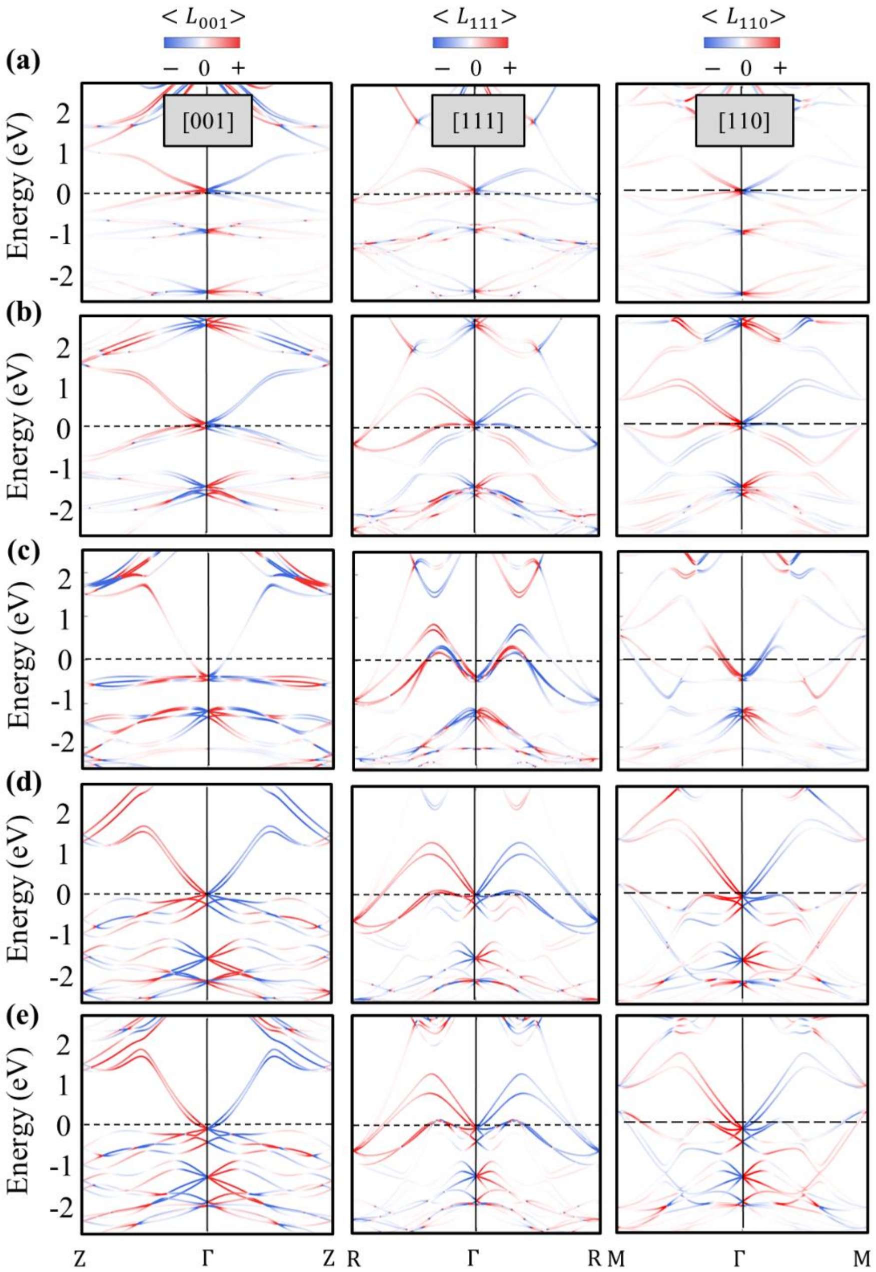

Fig. S1. OAM-resolved band structure. OAM of bulk electronic structures of **(a)** CoSi, **(b)** RhSi, **(c)** PdGa, **(d)** AlPt, and **(e)** PtGa along [001], [111], and [110] momentum directions in enantiomer A. For all these compounds, OAM reverses signs at $+k$ and $-k$.

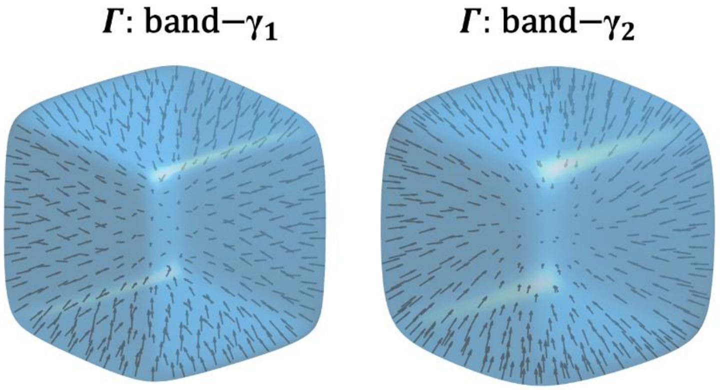

Fig. S2. Calculated 3D OAM texture for the spin-split FSs in PdGa. OAM texture for the spin-split FSs formed by the band γ_1 and γ_2 (as indicated in Fig. 1(c)) that centered at the Γ point.

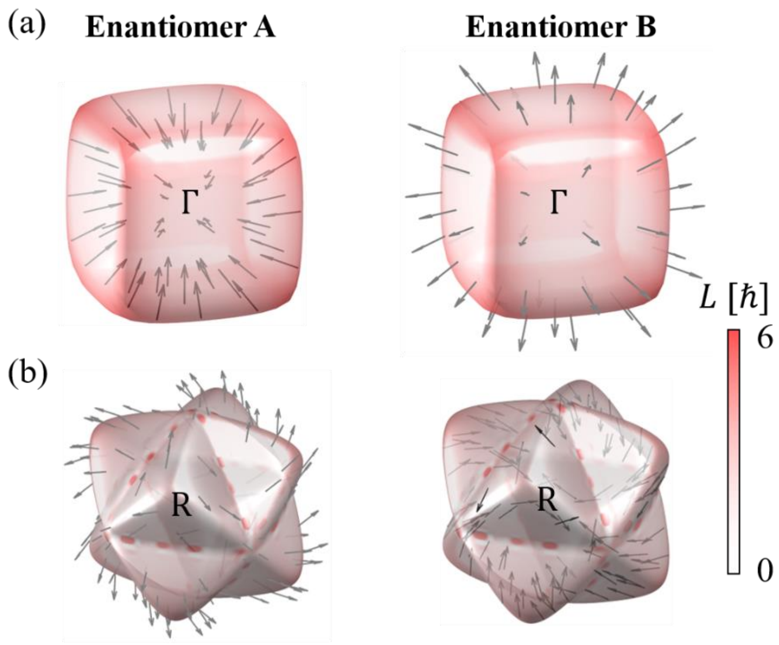

Fig. S3. OAM texture around chiral fermions in PdGa. Inward and outward OAM texture in PdGa enantiomers around chiral fermions at **(a)** Γ and **(b)** R point, respectively. OAM texture was calculated for the Fermi surface pockets formed by the band γ_2 centered at the Γ and R point, respectively, as indicated in Fig. 1(c).

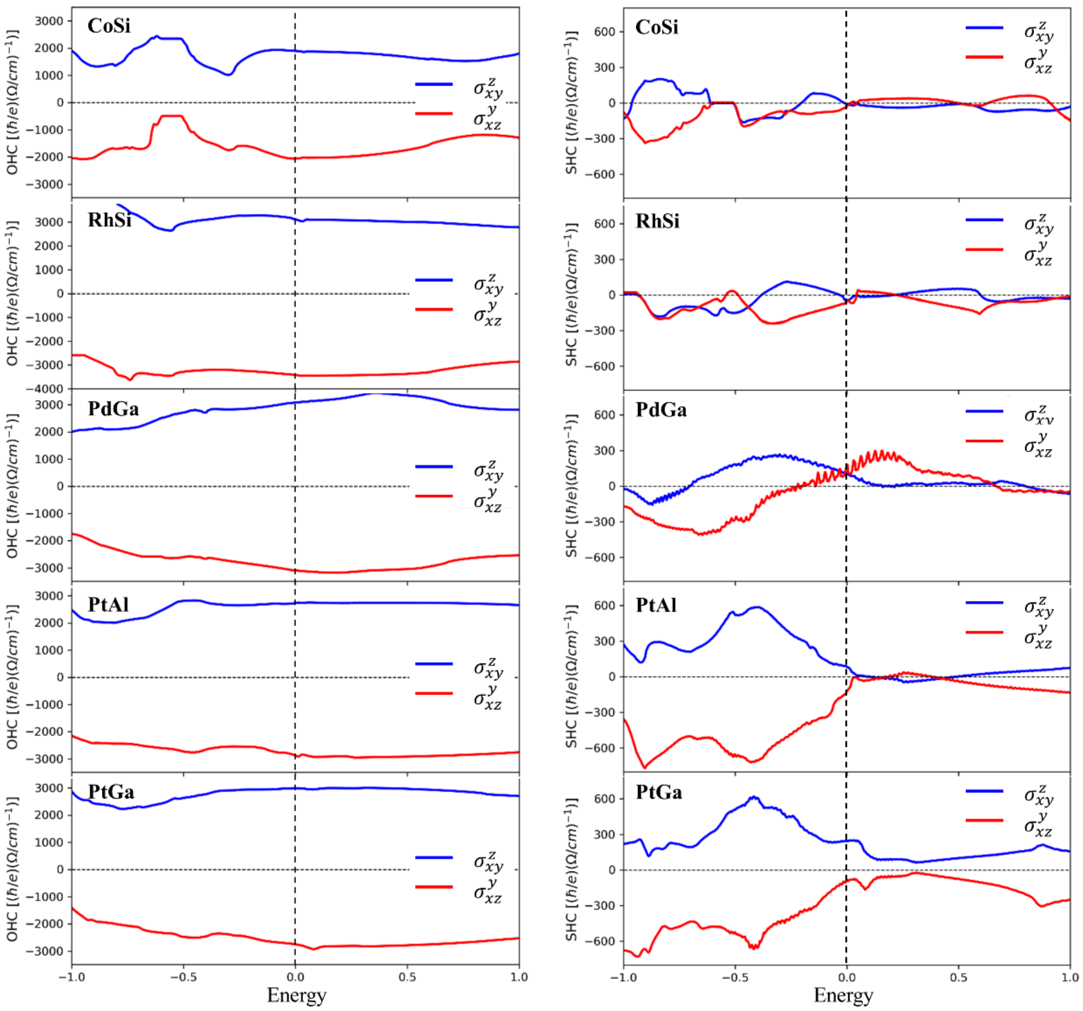

Fig. S4. Energy-dependent orbital Hall conductivity (OHC) and spin Hall conductivity (SHC). Energy-dependent OHC and SHC tensor elements σ_{xy}^{z} and σ_{xz}^{y} for CoSi, RhSi, PdGa, PtAl and PtGa. The Fermi energy is set to zero at the charge neutral point.

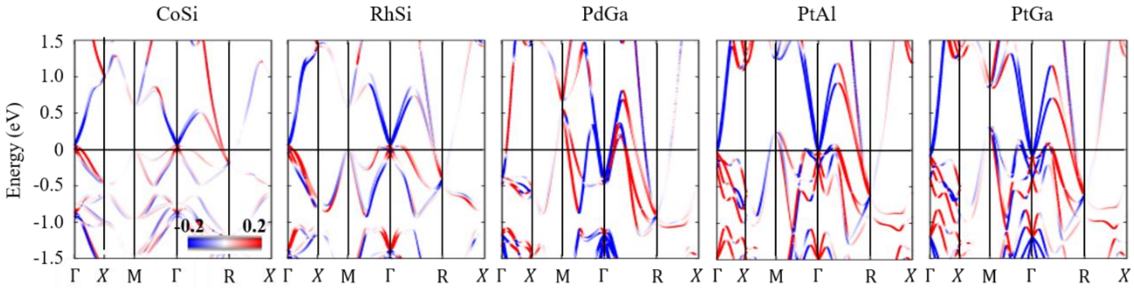

Fig. S5. Electric current-induced magnetization M_0-resolved band structure for enantiomers A of topological chiral semimetals. The local M_0 (k)-resolved band structures along high symmetry k path. The electric field is assumed to be $E_x = 10^5\ Vm^{-1}$.

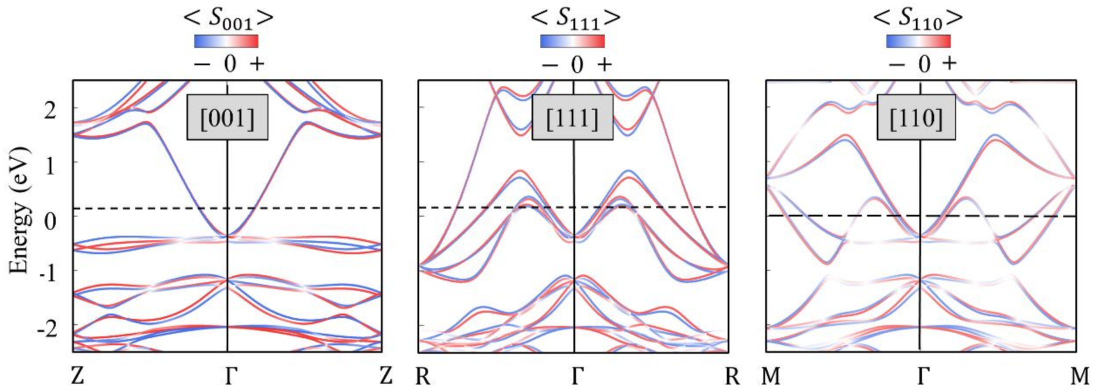

Fig. S6. SAM-resolved band structure in PdGa. SAM of bulk electronic structures along [001], [111], and [110] momentum directions in PdGa enantiomer A. The spin-split bands carry the antiparallel SAM.

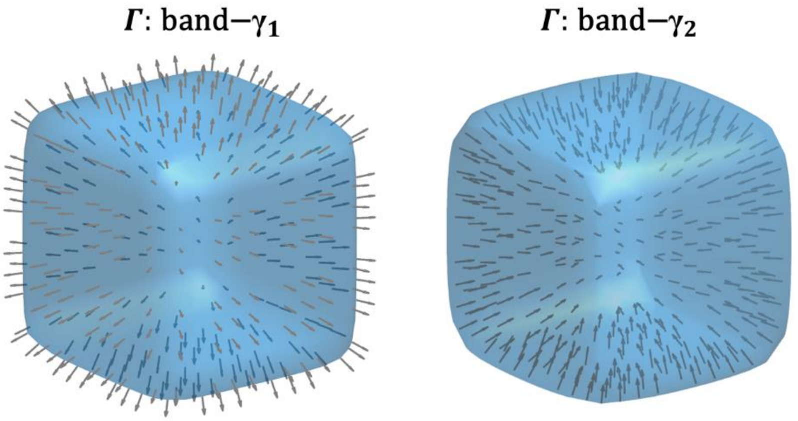

Fig. S7. Calculated 3D SAM texture for the spin-split FSs in PdGa. SAM texture for the spin-split FSs formed by the band γ_1 and γ_2 (as indicated in Fig. 1(c)) that centered at the Γ point.

Table S1. Symmetry properties of topological chiral semimetals. Symmetry relations of OAM denoted by L in k space for the generators of space group P$2_1$3.

Symmetry	x-component (L_x)	y-component (L_y)	z-component (L_z)
$\hat{T}$	$L_x(\boldsymbol{k}) \rightarrow -L_x(-\boldsymbol{k})$	$L_y(\boldsymbol{k}) \rightarrow -L_y(-\boldsymbol{k})$	$L_z(\boldsymbol{k}) \rightarrow -L_z(-\boldsymbol{k})$
$\mathbf{2_{1x}}$	$L_x(k_x, k_y, k_z) \rightarrow L_x(k_x, -k_y, -k_z)$	$L_y(k_x, k_y, k_z) \rightarrow -L_y(k_x, -k_y, -k_z)$	$L_z(k_x, k_y, k_z) \rightarrow -L_z(k_x, -k_y, -k_z)$
$\mathbf{2_{1y}}$	$L_x(k_x, k_y, k_z) \rightarrow -L_x(-k_x, k_y, -k_z)$	$L_y(k_x, k_y, k_z) \rightarrow L_y(-k_x, k_y, -k_z)$	$L_z(k_x, k_y, k_z) \rightarrow -L_z(-k_x, k_y, -k_z)$
$\boldsymbol{C_3}$	$L_x(k_x, k_y, k_z) \rightarrow L_z(k_y, k_z, k_x)$	$L_y(k_x, k_y, k_z) \rightarrow L_x(k_y, k_z, k_x)$	$L_z(k_x, k_y, k_z) \rightarrow L_y(k_y, k_z, k_x)$

Table S2. OHC/SHC tensors for the chiral crystals in the 11 chiral point groups.

Point group	$\boldsymbol{\sigma^{x}_{\alpha\beta}}$	$\boldsymbol{\sigma^{y}_{\alpha\beta}}$	$\boldsymbol{\sigma^{z}_{\alpha\beta}}$
$\mathbf{C_1}$	$\begin{pmatrix} \sigma^x_{xx} & \sigma^x_{xy} & \sigma^x_{xz} \\ \sigma^x_{yx} & \sigma^x_{yy} & \sigma^x_{yz} \\ \sigma^x_{zx} & \sigma^x_{zy} & \sigma^x_{zz} \end{pmatrix}$	$\begin{pmatrix} \sigma^y_{xx} & \sigma^y_{xy} & \sigma^y_{xz} \\ \sigma^y_{yx} & \sigma^y_{yy} & \sigma^y_{yz} \\ \sigma^y_{zx} & \sigma^y_{zy} & \sigma^y_{zz} \end{pmatrix}$	$\begin{pmatrix} \sigma^z_{xx} & \sigma^z_{xy} & \sigma^z_{xz} \\ \sigma^z_{yx} & \sigma^z_{yy} & \sigma^z_{yz} \\ \sigma^z_{zx} & \sigma^z_{zy} & \sigma^z_{zz} \end{pmatrix}$
$\mathbf{C_2}$	$\begin{pmatrix} 0 & \sigma^x_{xy} & 0 \\ \sigma^x_{yx} & 0 & \sigma^x_{yz} \\ 0 & \sigma^x_{zy} & 0 \end{pmatrix}$	$\begin{pmatrix} \sigma^y_{xx} & 0 & \sigma^y_{xz} \\ 0 & \sigma^y_{yy} & 0 \\ \sigma^y_{zx} & 0 & \sigma^y_{zz} \end{pmatrix}$	$\begin{pmatrix} 0 & \sigma^z_{xy} & 0 \\ \sigma^z_{yx} & 0 & \sigma^z_{yz} \\ 0 & \sigma^z_{zy} & 0 \end{pmatrix}$
$\mathbf{C_3}$	$\begin{pmatrix} \sigma^x_{xx} & \sigma^x_{xy} & \sigma^x_{xz} \\ \sigma^x_{yx} & \sigma^x_{yy} & \sigma^x_{yz} \\ \sigma^x_{zx} & \sigma^x_{zy} & 0 \end{pmatrix}$	$\begin{pmatrix} \sigma^y_{xx} & \sigma^y_{xy} & \sigma^y_{xz} \\ \sigma^y_{yx} & \sigma^y_{yy} & \sigma^y_{yz} \\ \sigma^y_{zx} & \sigma^y_{zy} & 0 \end{pmatrix}$	$\begin{pmatrix} \sigma^z_{xx} & \sigma^z_{xy} & 0 \\ \sigma^z_{yx} & \sigma^z_{yy} & 0 \\ 0 & 0 & \sigma^z_{zz} \end{pmatrix}$
	$\sigma^x_{xx} = -\sigma^x_{yy} = -\sigma^y_{yx} = -\sigma^y_{xy};\ \sigma^y_{xx} = \sigma^x_{yx} = -\sigma^y_{yy} = \sigma^x_{xy};\ \sigma^x_{xz} = \sigma^y_{yz};$ $\sigma^x_{zx} = \sigma^y_{zy};\ \sigma^y_{zx} = -\sigma^x_{zy};\ \sigma^y_{xz} = -\sigma^x_{yz};\ \sigma^z_{xx} = \sigma^z_{yy};\ \sigma^z_{xy} = -\sigma^z_{yx}$		
$\mathbf{C_4}$	$\begin{pmatrix} 0 & 0 & \sigma^x_{xz} \\ 0 & 0 & \sigma^x_{yz} \\ \sigma^x_{zx} & \sigma^x_{zy} & 0 \end{pmatrix}$	$\begin{pmatrix} 0 & 0 & \sigma^y_{xz} \\ 0 & 0 & \sigma^y_{yz} \\ \sigma^y_{zx} & \sigma^y_{zy} & 0 \end{pmatrix}$	$\begin{pmatrix} \sigma^z_{xx} & \sigma^z_{xy} & 0 \\ \sigma^z_{yx} & \sigma^z_{yy} & 0 \\ 0 & 0 & \sigma^z_{zz} \end{pmatrix}$
	$\sigma^x_{zx} = \sigma^y_{zy};\ \sigma^x_{xz} = \sigma^y_{yz};\ \sigma^y_{zx} = -\sigma^x_{zy};\ \sigma^y_{xz} = -\sigma^x_{yz};\ \sigma^z_{xx} = \sigma^z_{yy};\ \sigma^z_{xy} = -\sigma^z_{yx}$		
$\mathbf{C_6}$	$\begin{pmatrix} 0 & 0 & \sigma^x_{xz} \\ 0 & 0 & \sigma^x_{yz} \\ \sigma^x_{zx} & \sigma^x_{zy} & 0 \end{pmatrix}$	$\begin{pmatrix} 0 & 0 & \sigma^y_{xz} \\ 0 & 0 & \sigma^y_{yz} \\ \sigma^y_{zx} & \sigma^y_{zy} & 0 \end{pmatrix}$	$\begin{pmatrix} \sigma^z_{xx} & \sigma^z_{xy} & 0 \\ \sigma^z_{yx} & \sigma^z_{yy} & 0 \\ 0 & 0 & \sigma^z_{zz} \end{pmatrix}$
	$\sigma^x_{zx} = \sigma^y_{zy};\ \sigma^x_{xz} = \sigma^y_{yz};\ \sigma^y_{zx} = -\sigma^x_{zy};\ \sigma^y_{xz} = -\sigma^x_{yz};\ \sigma^z_{xx} = \sigma^z_{yy};\ \sigma^z_{xy} = -\sigma^z_{yx}$		
$\boldsymbol{D_2}$	$\begin{pmatrix} 0 & 0 & 0 \\ 0 & 0 & \sigma^x_{yz} \\ 0 & \sigma^x_{zy} & 0 \end{pmatrix}$	$\begin{pmatrix} 0 & 0 & \sigma^y_{xz} \\ 0 & 0 & 0 \\ \sigma^y_{zx} & 0 & 0 \end{pmatrix}$	$\begin{pmatrix} 0 & \sigma^z_{xy} & 0 \\ \sigma^z_{yx} & 0 & 0 \\ 0 & 0 & 0 \end{pmatrix}$

$\boldsymbol{D_3}$ (SGs. 149, 151, 153)	$\begin{pmatrix} 0 & \sigma^x_{xy} & 0 \\ \sigma^x_{yx} & 0 & \sigma^x_{yz} \\ 0 & \sigma^x_{zy} & 0 \end{pmatrix}$ $\begin{pmatrix} \sigma^y_{xx} & 0 & \sigma^y_{xz} \\ 0 & \sigma^y_{yy} & 0 \\ \sigma^y_{zx} & 0 & 0 \end{pmatrix}$ $\begin{pmatrix} 0 & \sigma^z_{xy} & 0 \\ \sigma^z_{yx} & 0 & 0 \\ 0 & 0 & 0 \end{pmatrix}$	$\sigma^x_{xy} = \sigma^x_{yx} = \sigma^y_{xx} = -\sigma^y_{yy};\ \sigma^y_{xz} = -\sigma^x_{yz}; \sigma^y_{zx} = -\sigma^x_{zy};\ \sigma^z_{xy} = -\sigma^z_{yx}$
$\boldsymbol{D_3}$ (SGs. 150, 152, 154)	$\begin{pmatrix} \sigma^x_{xx} & 0 & 0 \\ 0 & \sigma^x_{yy} & \sigma^x_{yz} \\ 0 & \sigma^x_{zy} & 0 \end{pmatrix}$ $\begin{pmatrix} 0 & \sigma^y_{xy} & \sigma^y_{xz} \\ \sigma^y_{yx} & 0 & 0 \\ \sigma^y_{zx} & 0 & 0 \end{pmatrix}$ $\begin{pmatrix} 0 & \sigma^z_{xy} & 0 \\ \sigma^z_{yx} & 0 & 0 \\ 0 & 0 & 0 \end{pmatrix}$	$\sigma^x_{xx} = -\sigma^x_{yy} = -\sigma^y_{yx} = -\sigma^y_{xy};\ \sigma^x_{zy} = -\sigma^y_{zx};\ \sigma^x_{yz} = -\sigma^y_{xz};\ \sigma^z_{xy} = -\sigma^z_{yx}$
$\boldsymbol{D_4}$	$\begin{pmatrix} 0 & 0 & 0 \\ 0 & 0 & \sigma^x_{yz} \\ 0 & \sigma^x_{zy} & 0 \end{pmatrix}$ $\begin{pmatrix} 0 & 0 & \sigma^y_{xz} \\ 0 & 0 & 0 \\ \sigma^y_{zx} & 0 & 0 \end{pmatrix}$ $\begin{pmatrix} 0 & \sigma^z_{xy} & 0 \\ \sigma^z_{yx} & 0 & 0 \\ 0 & 0 & 0 \end{pmatrix}$	$\sigma^y_{xz} = -\sigma^x_{yz};\ \sigma^x_{zy} = -\sigma^y_{zx};\ \sigma^z_{xy} = -\sigma^z_{yx}$
$\boldsymbol{D_6}$	$\begin{pmatrix} 0 & 0 & 0 \\ 0 & 0 & \sigma^x_{yz} \\ 0 & \sigma^x_{zy} & 0 \end{pmatrix}$ $\begin{pmatrix} 0 & 0 & \sigma^y_{xz} \\ 0 & 0 & 0 \\ \sigma^y_{zx} & 0 & 0 \end{pmatrix}$ $\begin{pmatrix} 0 & \sigma^z_{xy} & 0 \\ \sigma^z_{yx} & 0 & 0 \\ 0 & 0 & 0 \end{pmatrix}$	$\sigma^x_{yz} = -\sigma^y_{xz};\ \sigma^x_{zy} = -\sigma^y_{zx};\ \sigma^z_{xy} = -\sigma^z_{yx}$
**O**	$\begin{pmatrix} 0 & 0 & 0 \\ 0 & 0 & \sigma^x_{yz} \\ 0 & \sigma^x_{zy} & 0 \end{pmatrix}$ $\begin{pmatrix} 0 & 0 & \sigma^y_{xz} \\ 0 & 0 & 0 \\ \sigma^y_{zx} & 0 & 0 \end{pmatrix}$ $\begin{pmatrix} 0 & \sigma^z_{xy} & 0 \\ \sigma^z_{yx} & 0 & 0 \\ 0 & 0 & 0 \end{pmatrix}$	$\sigma^z_{xy} = \sigma^x_{yz} = \sigma^y_{zx} = -\sigma^z_{yx} = -\sigma^x_{zy} = -\sigma^y_{xz}$
**T**	$\begin{pmatrix} 0 & 0 & 0 \\ 0 & 0 & \sigma^x_{yz} \\ 0 & \sigma^x_{zy} & 0 \end{pmatrix}$ $\begin{pmatrix} 0 & 0 & \sigma^y_{xz} \\ 0 & 0 & 0 \\ \sigma^y_{zx} & 0 & 0 \end{pmatrix}$ $\begin{pmatrix} 0 & \sigma^z_{xy} & 0 \\ \sigma^z_{yx} & 0 & 0 \\ 0 & 0 & 0 \end{pmatrix}$	$\sigma^z_{xy} = \sigma^x_{yz} = \sigma^y_{zx};\ \sigma^z_{yx} = \sigma^x_{zy} = \sigma^y_{xz}$

Table S3. Current-induced magnetization in different materials. The current-induced spin and orbital magnetization for the topological chiral semimetals at the charge neutral point. For comparison, current-induced spin magnetization for the strong Rashba systems Au (111) and Bi/Ag (111), the topological insulator α-Sn (001) and current-induced orbital magnetization for the strained twisted bilayer graphene (TBG) are also listed. It is noted that the values of current-induced magnetization in topological chiral semimetals were unified in the unit of μ_B/nm^2 for comparison which was obtained by $M_0 \times a$, where a is the lattice constant. The corresponding bulk magnetization with the units of μ_B/nm^3 is also listed in the bracket. τ is the relaxation time. The electric field is assumed to be $E_x = 10^5\ Vm^{-1}$.

**System**	**τ** (ps)	**Spin magnetization** μ_B/nm^2	**Orbital magnetization** μ_B/nm^2
Au(111) (2–4)	0.26	1.5$\times 10^{-3}$	~
Bi/Ag(111) (2, 3, 5)	0.31	10.3×10^{-3}	~
$\alpha - Sn(001)$ (2, 6)	0.39	5.7$\times 10^{-3}$	~
Strained TBG (7)	10	~	0.20
CoSi	1.0	-2.88$\times 10^{-4}$(-6.5 $\times 10^{-4}$ μ_B/nm^3)	0.028(0.064 μ_B/nm^3)
RhSi	1.0	-1.64$\times 10^{-3}$(-3.5$\times 10^{-3}$ μ_B/nm^3)	0.049(0.105 μ_B/nm^3)
PdGa	1.0	-1.81$\times 10^{-3}$(-3.7$\times 10^{-3}$ μ_B/nm^3)	0.038(0.081 μ_B/nm^3)
PtAl	1.0	4.29×10^{-4}(8.8$\times 10^{-4}$ μ_B/nm^3)	0.014(0.028 μ_B/nm^3)
PtGa	1.0	-2.21$\times 10^{-3}$(-4.5$\times 10^{-3}$ μ_B/nm^3)	-0.021(-0.039 μ_B/nm^3)

SI References

1. W.-Y. He, X. Y. Xu, K. T. Law, Kramers Weyl semimetals as quantum solenoids and their applications in spin-orbit torque devices. *Commun. Phys.* 4, 1–8 (2021).
2. A. Johansson, J. Henk, I. Mertig, Theoretical aspects of the Edelstein effect for anisotropic two-dimensional electron gas and topological insulators. *Phys. Rev. B* 93, 195440 (2016).
3. A. Johansson, J. Henk, I. Mertig, Edelstein effect in Weyl semimetals. *Phys. Rev. B* 97, 085417 (2018).
4. S. LaShell, B. A. McDougall, E. Jensen, Spin Splitting of an Au(111) Surface State Band Observed with Angle Resolved Photoelectron Spectroscopy. *Phys. Rev. Lett.* 77, 3419–3422 (1996).
5. H. J. Zhang, et al., Charge-to-Spin Conversion and Spin Diffusion in Bi/Ag Bilayers Observed by Spin-Polarized Positron Beam. *Phys. Rev. Lett.* 114, 166602 (2015).
6. J.-C. Rojas-Sánchez, et al., Spin to Charge Conversion at Room Temperature by Spin Pumping into a New Type of Topological Insulator: α-Sn Films. *Phys. Rev. Lett.* 116, 096602 (2016).
7. W.-Y. He, D. Goldhaber-Gordon, K. T. Law, Giant orbital magnetoelectric effect and current-induced magnetization switching in twisted bilayer graphene. *Nat. Commun.* 11, 1650 (2020).
